# Biomechanical analysis of effective mandibular en-masse retraction using Class II elastics with a clear aligner: a finite element study

**DOI:** 10.1186/s40510-022-00417-4

**Published:** 2022-07-11

**Authors:** Qiuyu Wang, Danni Dai, Jianrong Wang, Yanfang Chen, Chao Zhang

**Affiliations:** Stomatological Hospital, Southern Medical University, Guangzhou, 510280 China

**Keywords:** Clear aligners, Finite element method, Class II elastics

## Abstract

**Background:**

This study aimed to evaluate the displacement and stress distribution of mandibular dentition by various positions of the Class II elastics during en-masse retraction in clear aligner therapy.

**Methods:**

Models including a mandibular dentition (without first premolars), periodontal ligament (PDL), mandible, as well as attachments, aligners and buttons were constructed and imported into Ansys Workbench 2019 (ANSYS, USA) to generate the three-dimensional (3D) finite element model. Six combinations were created: (1) aligner alone (control), (2)-(5) Class II elastics with buttons placed on the mesiobuccal (MB), distobuccal (DB), mesiolingual (ML) and distolingual (DL) surface of the mandibular first molar, and (6) Class II elastics with a button on the aligner corresponding to the mesiobuccal surface of the mandibular first molar (AMB). The elastic force was set to 2 N for simulations.

**Results:**

The central incisors appeared lingual tipping in the six models. The lingual crown movement of the central incisors was 0.039 mm, 0.034 mm, 0.034 mm, 0.042 mm, 0.041 mm, and 0.034 mm for control model, MB model, DB model, ML model, DL model, and AMB model, respectively. The first molars showed mesial tipping in the six models. The mesial movement of the mesiobuccal cusps of the first molars was 0.045 mm, 0.060 mm, 0.063 mm, 0.048 mm, 0.051 mm, and 0.055 mm for control model, MB model, DB model, ML model, DL model, and AMB model, respectively.

**Conclusions:**

Class II elastics reduced lingual tipping of anterior teeth but aggravated mesial tipping of posterior teeth. Mesiolingual elastics developed minimum mesial tipping of the posterior teeth. When Class II elastics are required, attaching elastics on the mesiolingual surface of the mandibular first molar is recommended to prevent mandibular anchorage loss.

## Background

The demand for clear aligner (CA) therapy has increased dramatically thanks to its advantages of aesthetics, therapeutic comfort, and engagement of new biomaterial [1]. It has been reported that the number of clinical cases for the treatment of malocclusion via CA is continuously growing and approaching to the number of cases treated by conventional technique, fixed appliances (FA) [2, 3]. However, it is suggested that CA therapy is mainly performed for non-extraction cases with minimal root movement [4–6]. Therapeutic outcomes of CA in four first premolar extraction cases which require large root movement remain unfavorable and thus become a critical challenge for dentists [7].

**Fig. 1:**
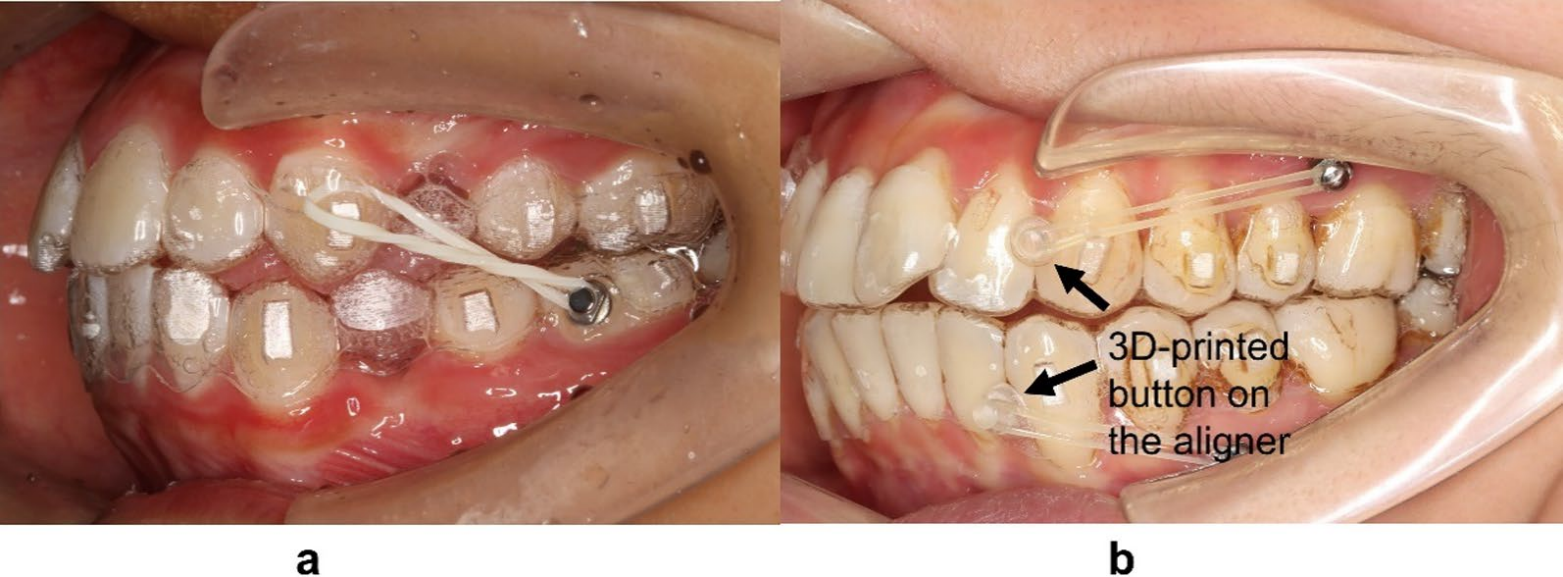
**a** Class II elastics engaged on the mesiobuccal surface of the mandibular first molar. **b** The 3D-printed button on the aligner

Dai et al. [8] reported unfavorable outcomes of the treatment with CA for four first premolar extraction cases which presented insufficient retraction of anterior teeth and excessive mesial displacements of the maxillary first molars. Machado [9] also concluded that an undesirable Class II relationship was found in the final occlusion in many CA cases with the involvement of tooth extraction. This was likely due to the minor loss in maxillary anchorage during anterior retraction. Therefore, the aligners system alone needs further improvement to treat extraction cases. That is why the auxiliary forces are so vital. Many researchers proposed to use Class II elastics via CA to reinforce anchorage and achieve Class I relationship, even in Class I extraction cases [8, 9] (Fig. 1a).

Aside from better anchorage control and the avoidance of mesial movement of the maxillary posterior teeth, Class II elastics may also produce the bowing effects in the maxillary arch. It is reported that the sagittal force component of Class II elastics improved the distalization of the maxillary canine, while the vertical force component of Class II elastics extruded the maxillary incisors [9, 10]. However, the role of Class II elastics in extraction cases on mandibular dentition has not yet been investigated. In FA therapy, Class II elastics can protract the mandibular posterior teeth via a pulling force, but the extrusion and clockwise rotation of the mandible are still present [11]. These effects have not been investigated in CA therapy with the engagement of Class II elastics. Further, the elastics are usually equipped on the buccal tubes of the mandibular molars in FA therapy [12, 13], while in CA therapy, different technical approaches could be equipped by applying the button on the buccal or lingual surface of the first molar [14, 15], or attached on a 3D-printed button on an aligner (Fig. 1b).

Finite element analysis (FEA), a noninvasive and accurate method, provides an approximate solution for the response of a geometric solid subjected to external forces [16]. Therefore, the FEA of a CA therapy could provide a better understanding of various orthodontic cases [17]. The aim of this study was to evaluate the displacement and stress distribution of mandibular dentition by various Class II elastics placements during en-masse retraction in CA therapy.

## Methods

The study protocol was approved by the Ethical Committee of Stomatological Hospital of Southern Medical University (SHSMU2021YW25).

### Model creation

A cone-beam computed tomography (CBCT) scan of an adult mandible with Class II division 1 was used for the study. The raw volumetric DICOM data from the scan was imported into Mimics 20.0 (Materialize Software, Leuven, Belgium) to create a 3D geometric model of the mandible and dentition, which was further improved by using Geomagic Studio 2014 (3D System, USA). The PDL was molded on the outer surface of the root with a uniform thickness of 0.25 mm in line with published studies [18]. The thickness of the cortical bone shell was set as 2 mm [19]. The mandibular right first premolar and its PDL were extracted to acquire the extraction dentition model.

The buttons, vertical rectangular attachments which are 3 mm in height, 2 mm in width, 1 mm in thickness, and horizontal rectangular attachments which are 2 mm in height, 3 mm in width, 1 mm in thickness, were modeled by using NX11 (Siemens, Germany) according to the clinical situation. The aligner was developed making an external offset from the crowns and attachments with a uniform thickness of 0.5 mm, in accordance with recent literature standards [16, 20, 21]. The vertical rectangular attachments were mounted on the center of the clinical crown of the canine and second premolar. The horizontal rectangular attachments were attached on the mesial buccal surface of the second molar as the retention point of the aligner. Six different models were developed based on the varied positions of the buttons and the horizontal rectangular attachments of the first molar.

The six models were including.

**Fig. 2:**
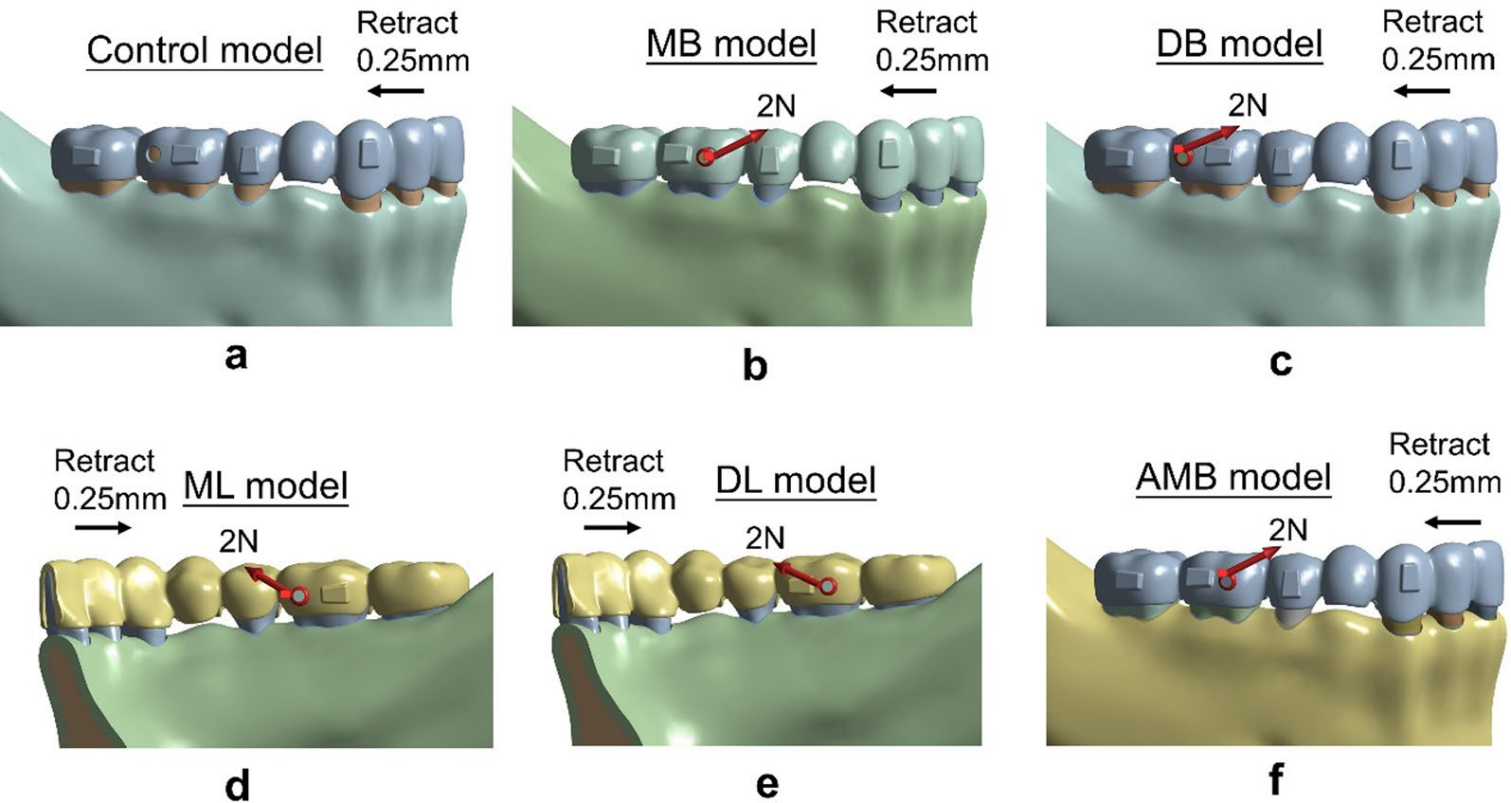
Six models. **a** The control model, **b** the MB model, **c** the DB model,** d** the ML model,** e** the DL model, and **f** the AMB model

- Control model—a mesiobuccal attachment (Fig. 2a).
- MB model—a distobuccal attachment and a mesiobuccal button (Fig. 2b).
- DB model—a mesiobuccal attachment and a distobuccal button (Fig. 2c).
- ML model—a distolingual attachment and a mesiolingual button (Fig. 2d).
- DL model—a mesiolingual attachment and a distolingual button (Fig. 2e).
- AMB model—a distobuccal attachment and a button on the aligner corresponding to the mesiobuccal surface of the tooth (Fig. 2f).

**Fig. 3:**
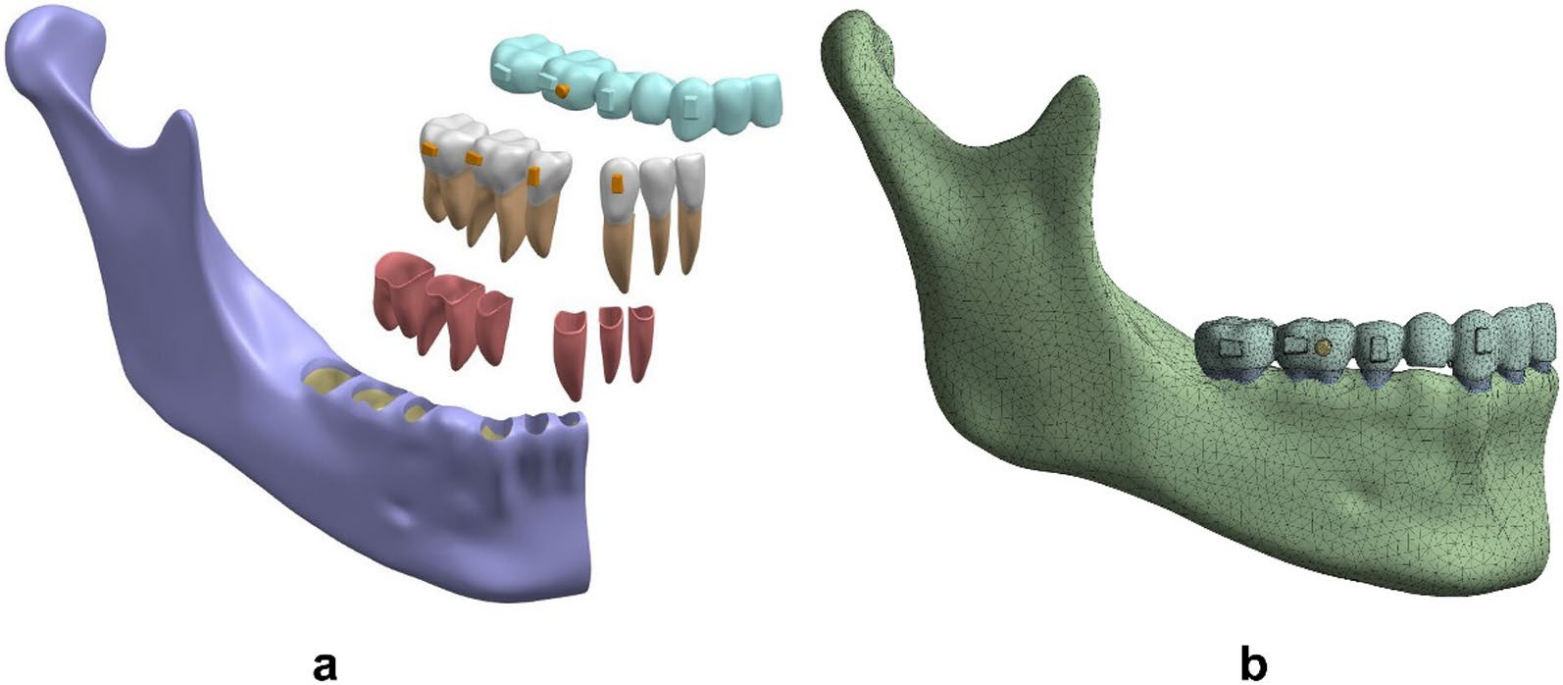
**a** Geometric model assembly of the aligner, teeth, PDL, mandible, attachments and the button. **b** the 3D finite element model

The components of each model were assembled and imported into Ansys Workbench 2019 (ANSYS, USA) to create a 3D finite element model (Fig. 3a, b). Each model was meshed as ten-noded tetrahedral elements. Mesh sizes for each component were set as follows: 1.0 mm for the dentition, 0.5 mm for the PDL, 2 mm for the mandibular bone, 0.5 mm for the attachments and buttons, and 1.0 mm for the aligners. Table 1 gave the number of elements and nodes for each model.

**Table 1:** Number of nodes and elements

Model	Number of nodes	Number of elements
Control	316,473	180,210
MB	318,537	181,398
DB	317,741	180,914
ML	317,697	180,907
DL	317,597	180,573
AMB	318,537	181,398

The material properties of all components were assumed to be linearly elastic, isotropic and homogeneous. Young’s modulus and Poisson's ratio were set in each component in terms of published experimental studies, as shown in Table 2 [17, 19, 20, 22–30].

**Table 2:** Material properties

Component	Young’s modulus (MPa)	Poisson’s ratio	References
Cancellous bone	1370	0.30	[17, 22]
Cortical bone	1.47 × 10^4^	0.30	[19, 23]
Tooth	1.96 × 10^4^	0.30	[20, 24]
PDL	0.143	0.45	[25]
Attachment	12.5 × 10^3^	0.36	[26]
Button	2.0 × 10^5^	0.33	[27]
CA	528	0.36	[26, 28–30]

### Boundary conditions

The bottom of the mandibular bone was set as fixed support. The connections between the first molar and the button, the teeth and the attachments, the teeth and PDL, PDL and alveolar bone were set to “Bonded.” The connections between the adjacent teeth were set to “No Separation.” “No separation” allows small amounts of frictionless sliding occur along contact faces. Surface contact elements with a Coulomb friction coefficient of 0.2 were created between the aligner and the crowns and attachments [20].

### Simulation of orthodontic tooth movement

**Fig. 4:**
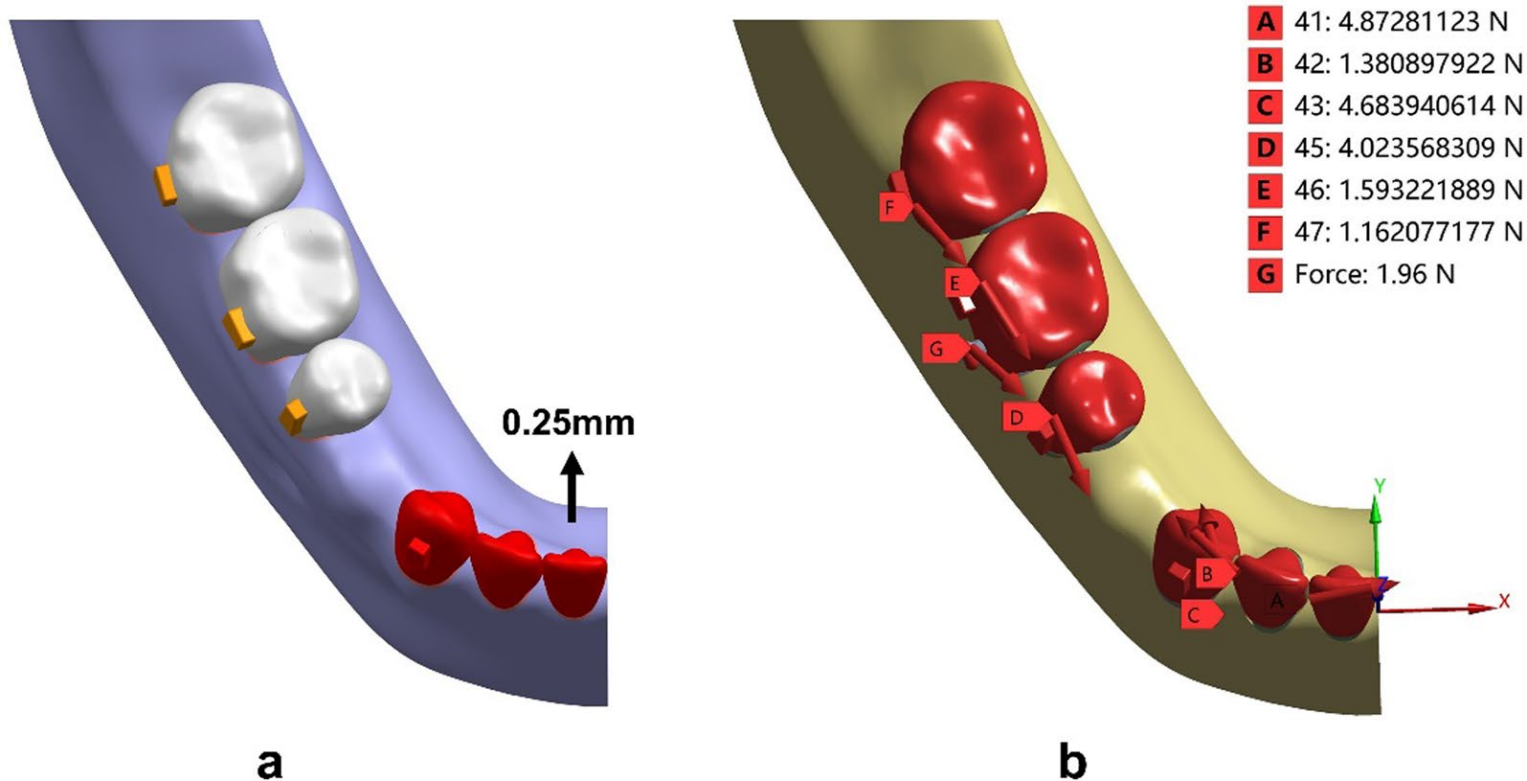
Loading method to simulate anterior en-masse retraction. **a** The displacement load was applied to the aligner by 0.25-mm lingual movement of the incisors and canine along the occlusal plane. **b** The upper right corner showed the force value of each tooth. A-H represented the forces exerted on the central incisor, the second incisor, the canine, the second premolar, the first molar and the second molar, respectively

The CA therapy employed the deformation of an aligner to yield orthodontic forces applied on the teeth. Six models were developed to mimic different therapeutic scenarios. The first control model simulated en-masse retraction of 0.25 mm via the aligner. In order to simulate the retraction force generated from the pre-tensioned aligner, lingual translation movements of the mandibular incisors and canine along the occlusal plane was set as 0.25 mm (Fig. 4a). The displacement load developed orthodontic forces via tensioned aligner exerting on the dentition (Fig. 4b).

The second to sixth models were created to represent different loading conditions by varying the positions of Class II elastics. The 2 N force was applied from the button on the mandibular first molar to the mesial cervical of the maxillary canine (Fig. 2b–f).

### Three-dimensional coordinate system

A three-dimensional coordinate system was established. The X-axis was defined as the mesio-distal direction, the Y-axis as the bucco-lingual direction, and the Z-axis as the occlusal-apical direction. A + X value was defined as the mesial direction, + Y as the lingual direction, and + Z as the occlusal direction. The central incisal edge and apex of the central incisor, cusp and apex of canine, buccal cusp and apex of second premolar, the mesiobuccal, distobuccal, mesiolingual cusps, and mesial apexes of the molars were taken as the measuring points. The aligner deformation, initial tooth displacement, principal stress of PDL, and von Mises stress of alveolar bone were analyzed. The region showing the maximum positive principal stress was regarded as the region of maximal tensile stress, and the region of minimum negative principal stress was considered as the region of maximum compressive stress.

## Results

### Aligner deformation

**Fig. 5:**
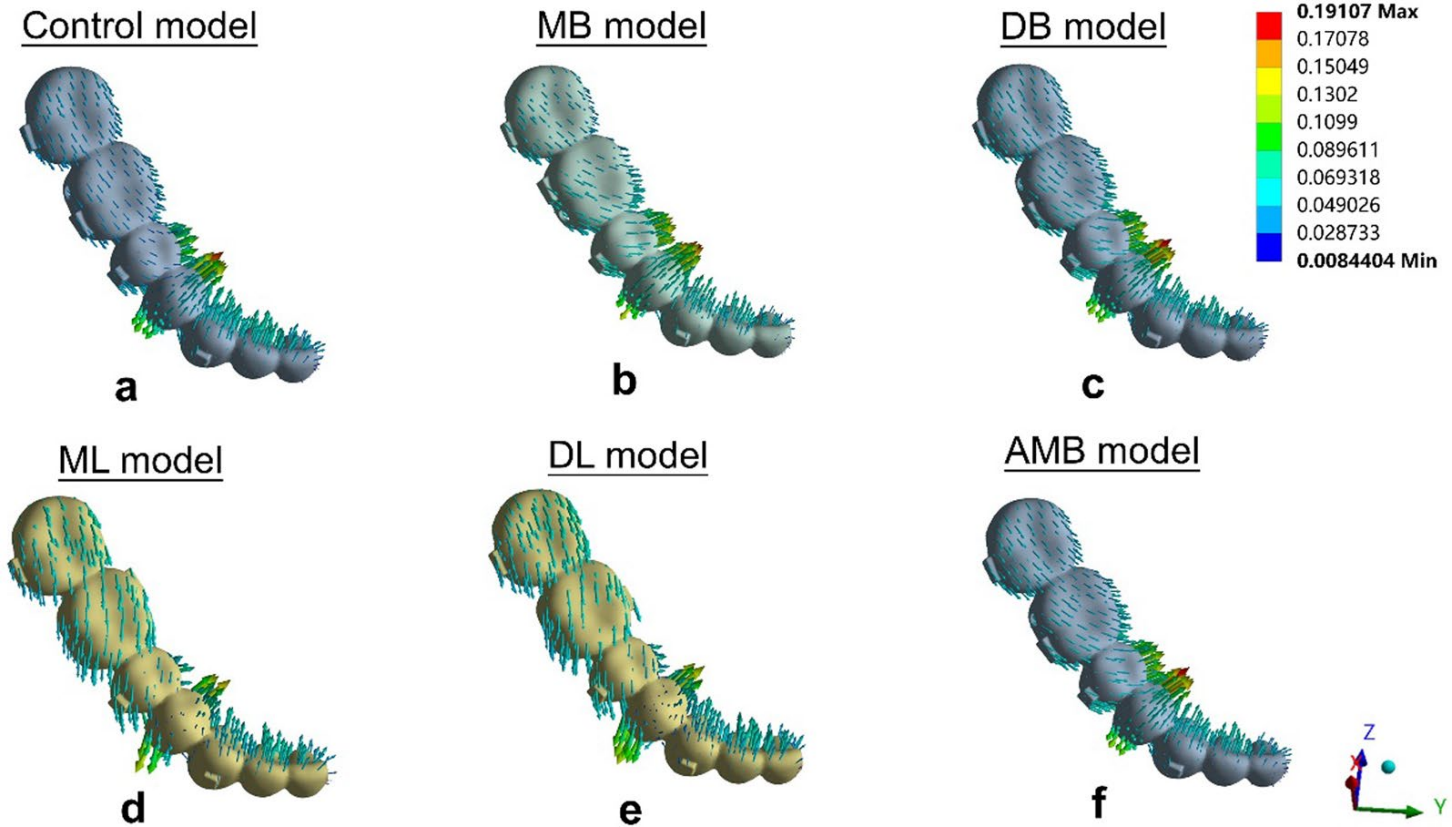
Tendencies of aligner deformation of **a** the control model, **b** the MB model, **c** the DB model,** d** the ML model,** e** the DL model, and **f** the AMB model

The aligner deformations are shown in Fig. 5 and Table 3. The maximum displacement of the aligner in the control model was located at the first premolar region (0.162 mm), suggesting a lingual protrusion tendency (Fig. 5a). The maximum aligner displacements in the buccal elastic models (MB model, DB model and AMB model) were also located at the first premolar region (0.191 mm in the MB model, 0.187 mm in the DB model and 0.187 mm in the AMB model). Figure 5b-c and 5f showed the lingual protrusion tendency. The maximum displacements of the aligners in the lingual elastic models (ML model and DL model) were lower than those in the control model (0.141 mm in the ML model and 0.142 mm in the DL model), and those located in the first premolar region where a labial-lingual protrusion could be triggered (Fig. 5d-e).

**Table 3:** Maximum aligner deformation and location

Aligner deformation	Max (mm)	Location
Control	0.162	L4 lingual cervical
MB	0.191	L4 lingual cervical
DB	0.187	L4 lingual cervical
ML	0.141	L4 buccal and lingual cervical
DL	0.142	L4 buccal and lingual cervical
AMB	0.187	L4 lingual cervical

L4: the mandibular first premolar

### Tooth displacement

**Fig. 6:**
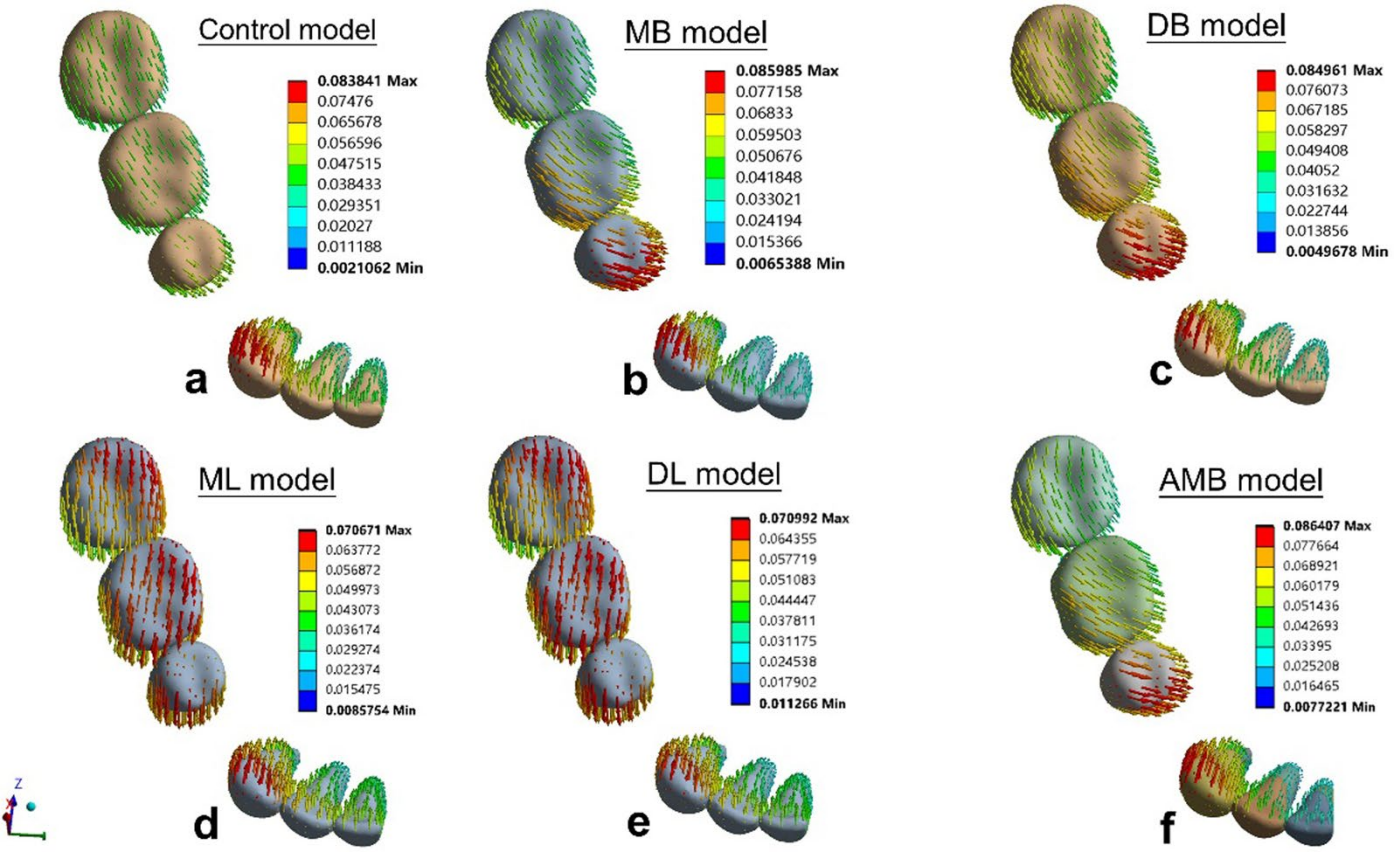
Displacement tendencies of the six models in the occlusal view. **a** The control model, **b** the MB model, **c** the DB model, **d** the MLmodel, **e** the DLmodel, and **f** the AMB model

**Fig. 7:**
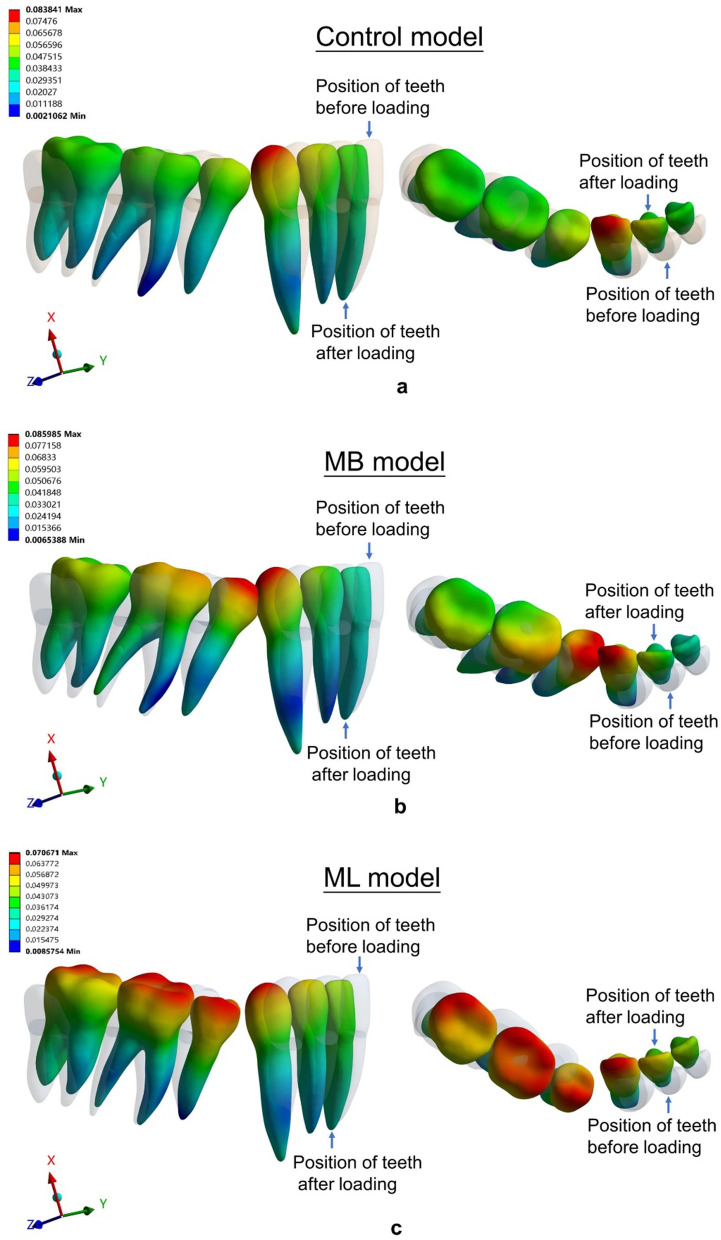
Displacements (mm) in three models: **a** the control model, **b** the MB model, and **c** the ML model. The gray contour shows the original position. The color image shows the initial displacement (× 80 times magnification)

The tooth displacements of the six simulations in three dimensions are shown in Tables 4, 5 and Figs. 6, 7.

**Table 4:** Displacements of the central incisor in the y-direction and of the canine in the x- and y-directions (mm)

	Axis	Control	MB	DB	ML	DL	AMB
C1	Y-axis	0.039	0.034	0.034	0.042	0.041	0.034
R1	Y-axis	0.019	0.019	0.019	0.019	0.019	0.018
C3	X-axis	− 0.027	− 0.024	− 0.024	− 0.027	− 0.026	− 0.025
	Y-axis	0.075	0.077	0.073	0.058	0.057	0.077
R3	X-axis	0.008	0.010	0.008	0.004	0.004	0.010
	Y-axis	− 0.029	− 0.030	− 0.029	− 0.021	− 0.020	− 0.031

X: + mesial, -distal; Y: + lingual, -buccal; Z: + occlusal, -apical; C1: the central incisal edge; R1: the apex of the central incisor; C3: the cusp of the canine; R3: the apex of the canine

**Table 5:** Displacement of posterior teeth in the X-, Y- and Z-directions (mm)

	Axis	Control	MB	DB	ML	DL	AMB
BC5	X-axis	0.047	0.060	0.063	0.053	0.055	0.058
	Y-axis	0.021	0.057	0.050	− 0.035	− 0.031	0.057
	Z-axis	− 0.019	− 0.019	− 0.024	− 0.023	− 0.026	− 0.017
R5	X-axis	0.002	− 0.010	− 0.011	− 0.001	− 0.004	− 0.006
	Y-axis	− 0.020	− 0.031	− 0.029	− 0.000	− 0.001	− 0.036
	Z-axis	− 0.016	− 0.017	− 0.020	− 0.010	− 0.013	− 0.016
MBC6	X-axis	0.045	0.060	0.063	0.048	0.051	0.055
	Y-axis	0.013	0.038	0.036	− 0.031	− 0.029	0.039
	Z-axis	− 0.005	0.011	0.006	− 0.021	− 0.023	0.012
DBC6	X-axis	0.047	0.062	0.066	0.052	0.056	0.058
	Y-axis	0.008	0.027	0.021	− 0.033	− 0.033	0.032
	Z-axis	0.005	0.022	0.019	− 0.006	− 0.006	0.022
MLC6	X-axis	0.040	0.042	0.046	0.057	0.058	0.043
	Y-axis	0.016	0.046	0.040	− 0.037	− 0.035	0.046
	Z-axis	− 0.011	− 0.010	− 0.012	− 0.002	− 0.004	− 0.010
MR6	X-axis	0.001	0.002	− 0.004	− 0.002	− 0.006	0.002
	Y-axis	− 0.002	− 0.020	− 0.017	0.026	0.027	− 0.019
	Z-axis	− 0.001	0.007	0.005	0.001	0.000	0.007
MBC7	X-axis	0.044	0.053	0.055	0.049	0.050	0.056
	Y-axis	0.004	0.015	0.011	− 0.018	− 0.017	0.009
	Z-axis	0.009	0.018	0.016	0.009	0.010	0.014
DBC7	X-axis	0.043	0.049	0.052	0.052	0.053	0.051
	Y-axis	− 0.003	0.002	− 0.001	− 0.014	− 0.012	− 0.010
	Z-axis	0.024	0.031	0.031	0.031	0.033	0.029
MLC7	X-axis	0.040	0.044	0.047	0.055	0.056	0.043
	Y-axis	0.004	0.017	0.013	− 0.020	− 0.018	0.011
	Z-axis	0.008	0.012	0.012	0.013	0.014	0.012
MR7	X-axis	0.005	0.007	0.007	0.008	0.007	0.006
	Y-axis	0.002	− 0.003	− 0.001	0.003	0.002	0.000
	Z-axis	0.016	0.020	0.021	0.024	0.025	0.020

X: + mesial, -distal; Y: + lingual, -buccal; Z: + occlusal, -apical; BC5: the buccal cusp of the second premolar; R5: the apex of the second premolar; MBC6: the mesiobuccal cusp of the mandibular first molar; DBC6: the distobuccal cusp of the first molar; MLC6: the mesiolingual cusp of the first molar; MR6: the mesial apex of the first molar; MBC7: the mesiobuccal cusp of the second molar; DBC7: the distobuccal cusp of the second molar; MLC7: the mesiolingual cusp of the second molar; MR7: the mesial apex of the second molar

The central incisors showed lingual crown tipping in the six models (Figs. 6, 7) Less lingual tipping occurred in the buccal elastic models (0.034 mm for MB model, DB model and AMB model, respectively) compared to that occurred in the control model (0.039 mm) (Table 4).

The canines exhibited lingual and distal crown tipping in the six models (Figs. 6, 7). Less lingual tipping was observed in the lingual elastic models (0.058 mm lingual crown movement for the ML model and 0.057 mm lingual crown movement for the DL model) compared to that in the control model (0.075 mm lingual crown movement) (Table 4). Less distal tipping was observed in the buccal elastic models (0.024 mm, 0.024 mm and 0.025 mm distal crown movement for MB, DB and AMB models, respectively) compared to that in the control model (0.027 mm distal crown movement) (Table 4).

In the anteroposterior (x-axis) dimension, the first molars showed mesial crown tipping in the six models (Figs. 6, 7). Greater mesial tipping was observed in the five elastic models (0.048–0.063 mm mesial crown movement) compared to that in the control model (0.045 mm mesial crown movement). The ML model showed the minimum mesial crown movement (0.048 mm), and the DB model showed the maximum mesial crown movement (0.063 mm) among the elastic models (Table 5).

In the transverse dimension (y-axis), the tooth displacement pattern of the first molars showed difference in the six models. Although lingual tipping with mesiolingual rotation occurred both in the control model and buccal elastic models (Figs. 6, 7), greater lingual tipping with mesiolingual rotation occurred in the buccal elastic models (0.038 mm, 0.036 mm and 0,039 mm lingual crown movement for MB, DB and AMB models, respectively) compared to that in the control model (0.013 mm) (Table 5). The lingual elastic models showed buccal tipping and distobuccal rotation of the crowns (0.031 mm,0.029 mm buccal crown movement of for ML and DL models, respectively) (Figs. 6, 7, Table 5). The tooth displacement patterns of the second premolars in the anteroposterior and transverse dimension were similar with that of the first molars.

In the vertical dimension (z-axis), intrusion of the second premolars was found in the six models (Fig. 7). The AMB model showed relatively low intrusion with 0.017 mm, compared with other models (Table 5). Extrusion of the second molars was found in the six models (Fig. 7). The control and ML models showed relatively low extrusion (0.009 mm) compared with other models (Table 5).

### Stress distribution

**Fig. 8:**
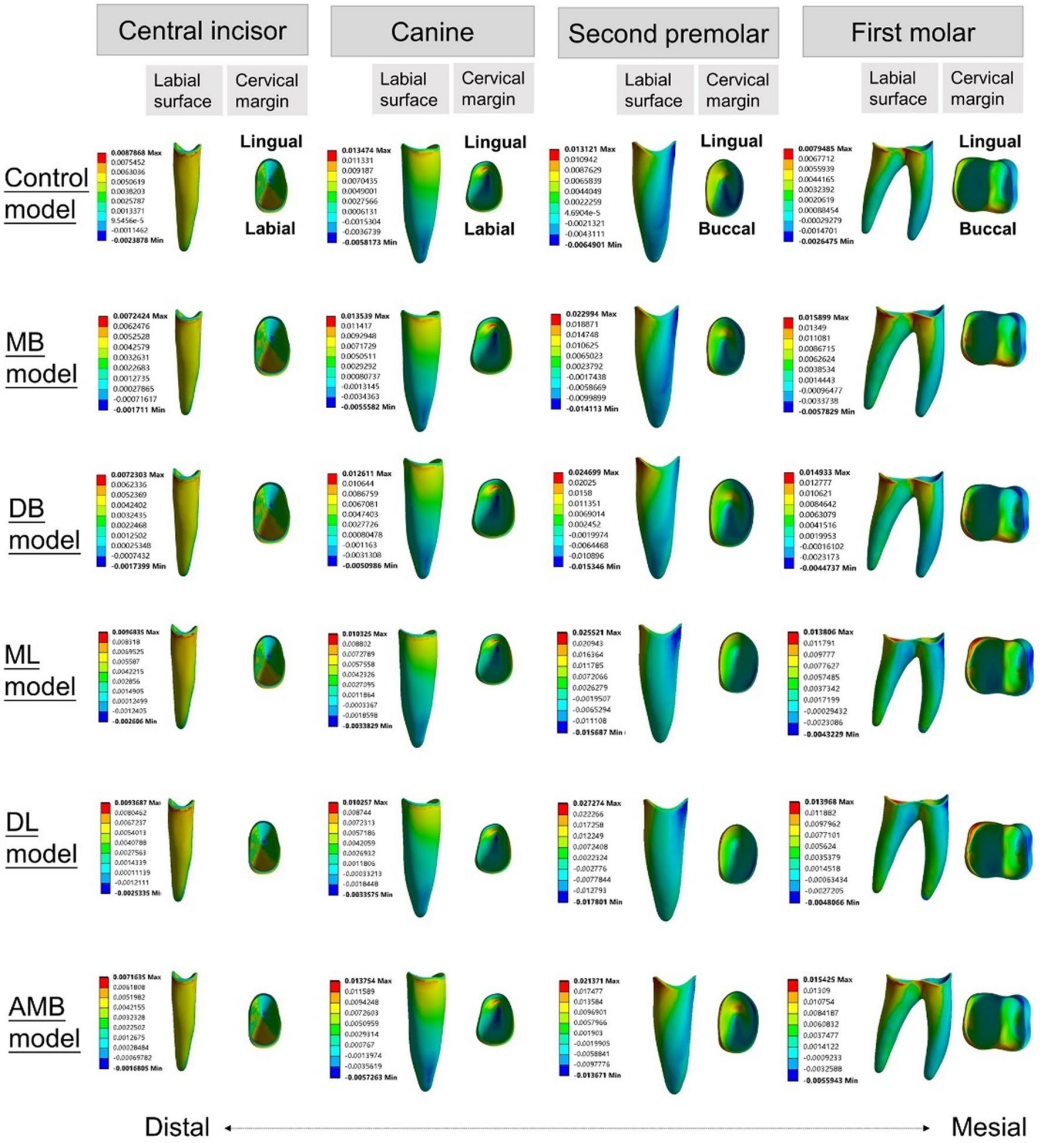
Maximum principal stress distribution of PDL in six models (MPa)

**Fig. 9:**
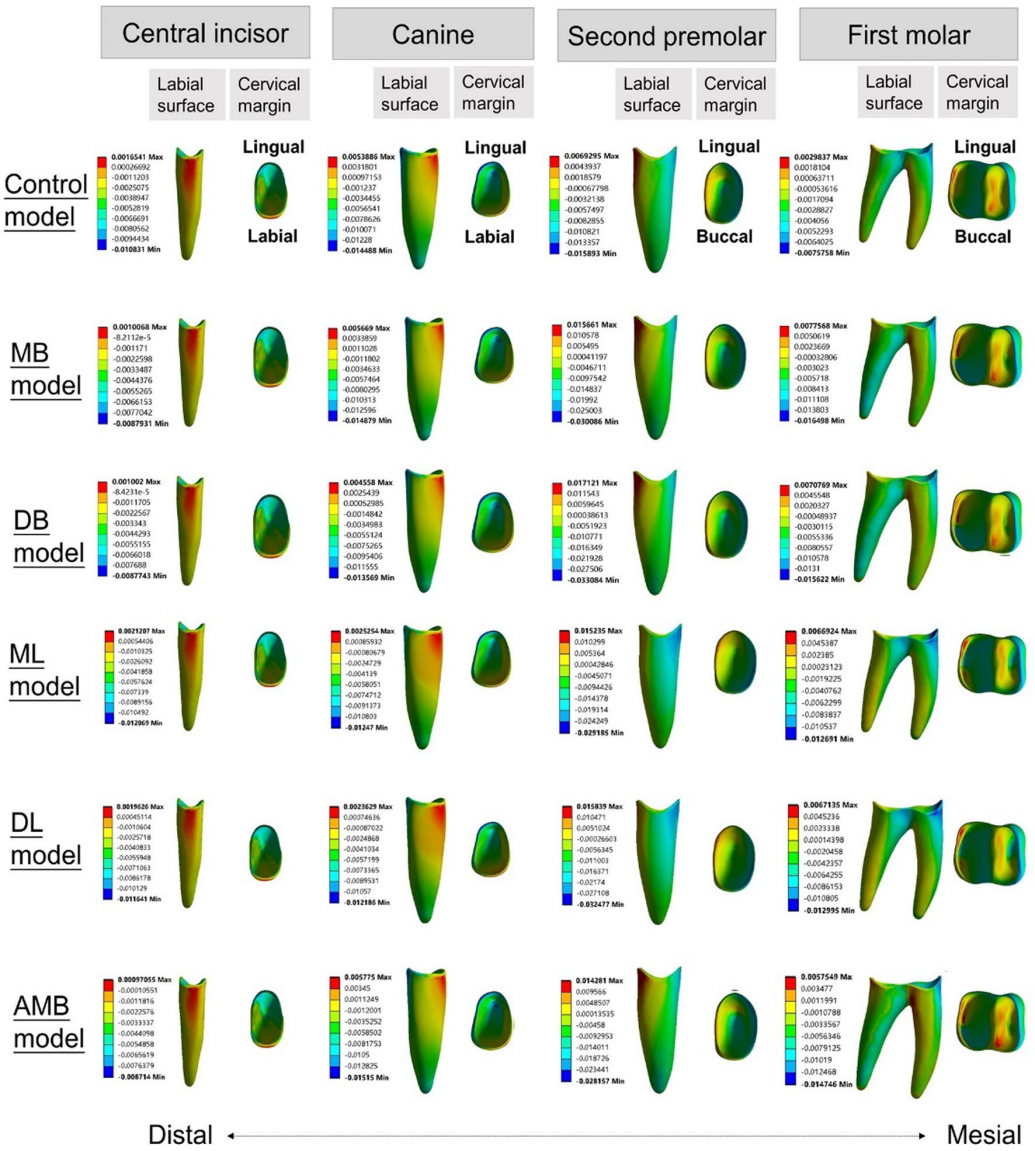
Minimum principal stress distribution of PDL in six models (MPa)

**Fig. 10:**
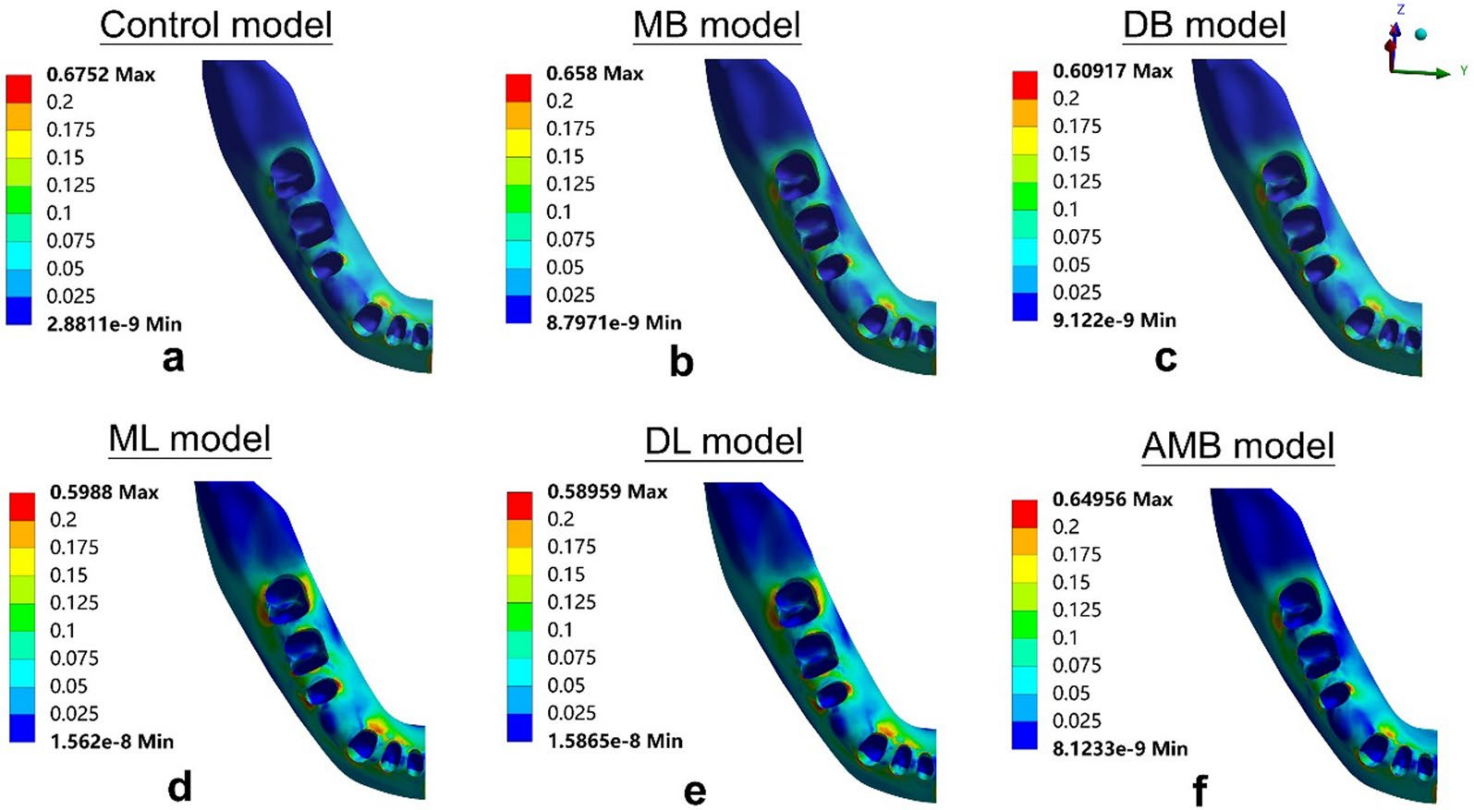
von Mises stress distribution of alveolar bone in six models (occlusal view): **a** the control model, **b** the MB model, **c** the DB model, **d** the ML model, **e** the DL model, and **f** the AMB model

The principal stresses of the PDL and the von Mises stresses of alveolar bone are shown in Figs. 8, 9, 10. For the stress on the PDL, the stress of all models was more likely to be concentrated in the cervical region of the PDL of the posterior teeth, especially in the second premolars (Figs. 8, 9). Significant compressive stress was present in the mesial cervical region of the PDL of the second premolars, and large tensile stress was present in the distal cervical region (Figs. 8, 9). For the first molars, there was apparent compressive stress in the mesial cervical region of the PDL, while there was significant tensile stress in the buccal-distal cervical region of the control model and buccal elastic models, and in the distal cervical region of the lingual elastic models (Figs. 8, 9).

In the elastic models, an increase in compressive and tensile stress of the posterior teeth was observed compared with the control model (Figs. 8, 9). The ML model produced lower stresses in the mesial and distal cervical region of the first molar PDL than the other four elastic models, with the maximum compressive stress of − 0.013 MPa and the maximum tensile stress of 0.014 MPa, respectively (Figs. 8, 9).

For the stress on alveolar bone, the stress concentration area in the control model was the lingual alveolar crest of the canine (0.675 MPa) (Fig. 10a). In the buccal elastic models, the stress concentrated in the lingual alveolar crests of the second premolars (0.609–0.658 MPa) (Fig. 10b-c, f). In the lingual elastic models, the stress concentrated in the buccal alveolar crests of the second premolars (0.590–0.599 MPa) (Fig. 10d-e). The maximum stress of alveolar bone in the control model exceeded that of all other models (Fig. 10a).

## Discussion

The 3D finite element method (FEM) for dental tissues and aligner generated in this study provided visual insight into investigating the biomechanical effects of mandibular anterior retraction during CA therapy. This approach further explored optimal strategies for Class II elastics management of malocclusion.

Several studies addressed the efficacy of CA therapy for en-masse retraction in maxillary dentition [20, 29]. It has been reported that during anterior retraction, CA therapy may result in lingual tipping and extrusion of incisors, while anterior mini-screws with elastics equipped on an aligner could achieve incisor intrusion and palatal root torquing [20]. Nowadays, there is no investigation of en-masse retraction in mandible. Therefore, the purpose of the present study was to analyze the displacement and stress distribution of mandibular dentition during en-masse retraction in CA therapy and reveal the underlying advantages and disadvantages of different positions for Class II elastics.

The control model without Class II elastics exhibited tipping movement rather than bodily movement. Lingual tipping of the central incisor, lingual and distal tipping of the canine were present in the control model, which is supported by Dai et al.’s study [31]. The mesial tipping of the first molar and tipping of the teeth adjacent to the extraction sites have been reported by Baldwin et al. [7]. Furthermore, the intrusion of the second premolar and extrusion of the second molar were also present in Zhu et al. [32]. These biomechanical features are similar to the bowing effect [33]. The advantages of Class II elastics are reflected in the torque control of the anterior teeth, while the disadvantages of Class II elastics are manifested in the poor control of the posterior teeth in the sagittal and vertical dimensions.

Class II elastics are conducive to torque control of the anterior teeth during en-masse retraction, as was observed in the displacement and stress distribution in the elastic models. Buccal elastics reduced the retroclination of incisors and lingual elastics prevented severe retroclination of canines. Moreover, Class II elastics reduced the distal tipping of the canines. These effects have clinical benefits for anterior retraction. Numerically, however, Class II elastics could only slightly improve the distal tipping of the canines. Thus, a preset angle of mesial crown tipping of the canines is recommended for clinical practices.

**Fig. 11:**
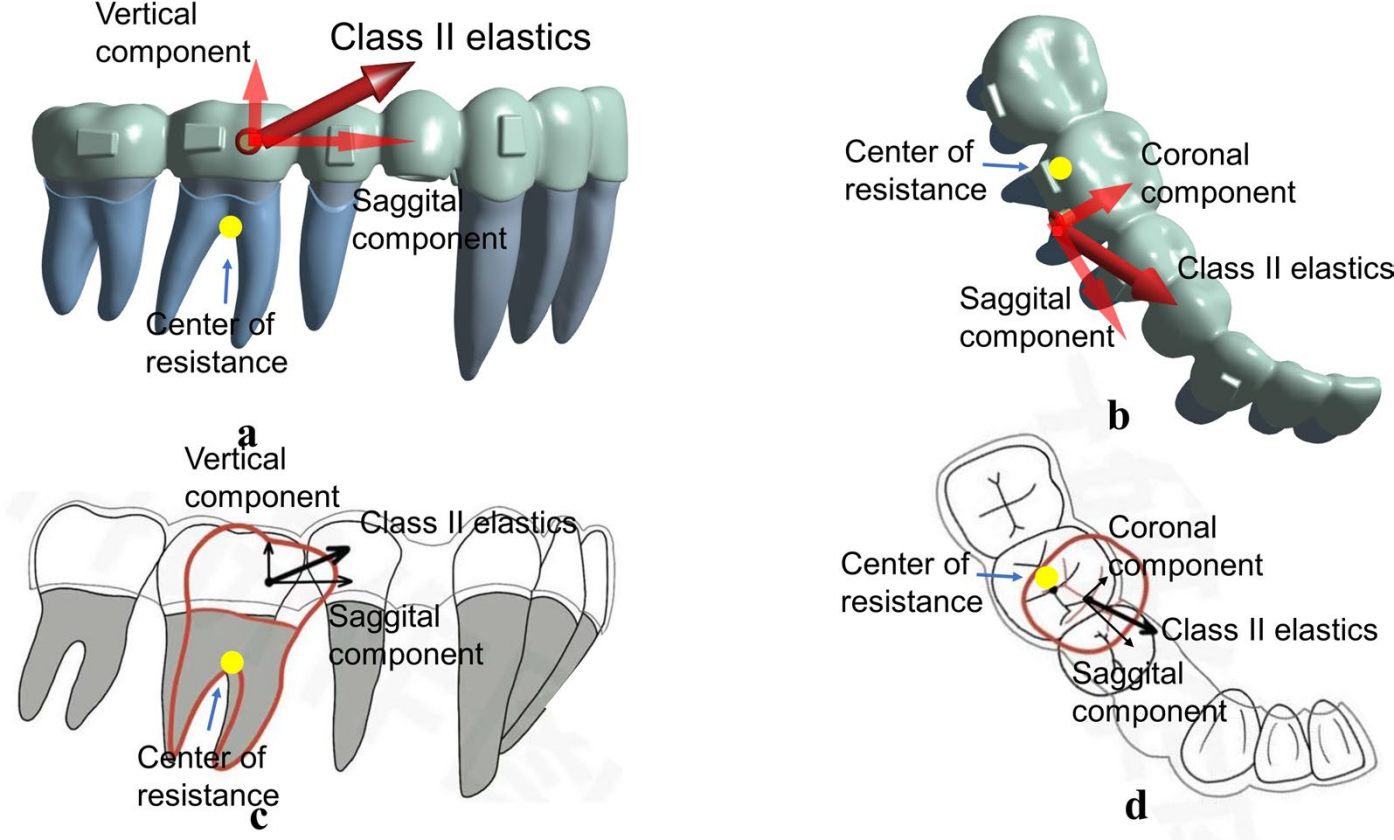
Elastics localization was located on the buccal side of the center of resistance of the lower first molar (**a, b**) which would aggravate the mesial tipping (**c**), lingual tipping and mesiolingual rotation (**d**) of the posterior teeth

However, Class II elastics are detrimental to the sagittal control of the anchorage for the posterior teeth. They may result in the mesial tipping of posterior teeth and posterior anchorage loss (Fig. 11a and c). Simulation results based on DB and DL models, where the elastics were attached on the distal surfaces, demonstrated a relatively more severe mesial tipping of posterior teeth compared with the MB and ML models, in which the elastics were attached on the mesial surfaces. The DB model is a common management strategy for the correction of Class II malocclusion because the caries or caries filling often happens to the mesiobuccal surface of the first molar and is not easy to bond the button. However, the worse performance of this approach on the prevention of mesial tipping is found in the simulation compared with other models developed in this study. The ML model exhibited the minimum mesial tipping of the posterior teeth among the elastic models. Mesiolingual elastics are recommended to minimize the excessive mesial tipping of the posterior teeth. Moreover, it is necessary to preset the distal tipping of the posterior teeth for anchorage preparation before retraction or elastics engagement. This is supported by Dai et al. [8] in which a distal tipping of 6.6° was recommended, as anchorage preparation effectively prevented the mesial tipping of the maxillary first molar before anterior retraction.

The buccal elastics produced lingual tipping of the first molar with mesiolingual rotation. The lingual elastics produced buccal tipping of the first molar with distobuccal rotation. The control model showed a minor lingual tipping of the first molar with mesiolingual rotation. The application of buccal elastics generated a buccal force that was exerted on the center of resistance of posterior teeth to increase the lingual tipping of posterior teeth (Fig. 11b and d). The tipping posterior teeth have relatively poor capability in keeping the root upright in alveolar bone and in carrying out transmission of occlusal forces [34]. Furthermore, the relatively large crown mesial-lingual rotation was present in posterior teeth, which might play a negative role for extraction treatment [34]. Thus, the recommendation is to expand the mandibular arch before engaging buccal elastics and to reduce the elastic force to prevent rotation. The engagement of lingual elastics placed on the first molar facilitates buccal tipping of the posterior teeth, which has clinical benefits on cases requiring more buccal inclination on posterior teeth. Furthermore, the buccal tipping of posterior teeth promotes molar protraction during space closure via root movement in cancellous bone [35]. In addition, distobuccal rotation of the posterior teeth can counteract the initial mesiolingual rotation and thus is beneficial to space closure [7].

Class II elastics do not work effectively at minimizing the bowing effects. The control model showed lingual tipping of the incisors, intrusion of the second premolar, and mesial tipping and extrusion of the second molar, which are consistent with the bowing effect in clinical manifestation [32, 33]. Janson et al. [10] concluded that the vertical force component of Class II elastics was able to extrude the maxillary incisors, which may produce the bowing effects in maxilla. The FEA shows that Class II elastics produced greater intrusion on the second premolar and greater mesial tipping and extrusion on the second molar and also aggravated the bowing effects in mandible. Therefore, a preset distal tipping of the molars and vertical elastics on the second premolar may be able to minimize the bowing effects.

Elastic forces generated from the intermaxillary elastics exerted on the buccal side of the aligner, as shown in the AMB model, can cause lingual deformation of the aligner. In comparison with the MB model, the deformed aligner yielded elastic forces to induce lingual tipping of the first molar and more mesiolingual rotation of the second molar. The AMB model represents a 3D-printed assembly of the button and aligner that has a clinical benefit regarding the prevention of gingival redness and swelling caused by poor hygiene of the periodontal tissues surrounding the button, which is widely used in clinical practice. However, as the button is attached on the aligner, the local deformation of the aligner induced by local forces exerted on the button is likely to cause uncontrolled movement of the tooth.

The stress distribution of the cervical region of the PDL was in line with the displacement tendency of the teeth. In the control model, the compressive stress was apparent in the mesial cervical region of the second premolar PDL, and the tensile stress was concentrated at the lingual surface (apical third) of the canine PDL. This was consistent with the phenomenon that tipping of the teeth adjacent to premolar extraction sites. The ML model produced the lowest stresses in the mesial and distal cervical region of the first molar PDL among the five elastic models, which was consistent with the displacement tendency of the first molar with the minimum mesial tipping in the elastic models.

The maximum stress present on the alveolar bone was 0.675 MPa in the control model, which is much lower than the ultimate tensile strength of the alveolar bone [36]. Hence, the alveolar bone in all models was safe during the en-masse retraction and the application of intermaxillary elastics. In addition, simulation results showed that the stress of alveolar bone was more evenly distributed in the lingual elastic models than in the control model and buccal elastic models, suggesting that lingual elastics could reduce the possibility of alveolar bone defects.

Some limitations should be mentioned in this study. First, the material properties of the aligners were simplified. In this study, the aligners were assumed to be linearly elastic and homogeneous, which was taken into account for two points. One point was that the aligner deformations were small enough to conform to Hooke's law, which could be approximately simulated with linearly elastic. The other point was that we hoped that our simulation results could be compared with other published studies to further investigate the biomechanical effects of anterior retraction during CA therapy, so the properties of the aligners were referred to other published studies, and the FEM was kept unified with other studies [20, 26, 28, 29]. However, the material properties of the aligners are actually polyurethane. Biphasic or poroelastic models on FEA can simulate tooth movement by the aligners more realistically. Second, the FEM results simply explained the initial effects of stress and tooth displacement in the PDL space prior to bone remodeling in one condition, and subsequent clinical results may not be similar to the initial response [27]. Third, the biomechanical effects of the attachment position on the mandibular dentition were not investigated in this study. Some studies have reported that attachment positions and shapes were able to develop mechanical torque exerted on teeth and may result in the displacement of teeth [37, 38]. Evaluation of biomechanical effects in other variations, such as different attachment positions or different attachment designs, may result in different outcomes.

## Conclusions

In summary, the FEM for mandibular dentition with CA in this study reveals significant biomechanical features related to clinical outcomes. En-masse retraction of mandibular dentition using CA produces lingual tipping of anterior teeth, tipping of the teeth adjacent to the extraction sites and mesial tipping of posterior teeth.

Class II elastic are conducive in torque control of anterior teeth. However, they aggravate the mesial tipping of posterior teeth and distal elastics can worsen mesial tipping. Along the same lines, buccal elastics tip the posterior teeth lingually with mesiolingual rotation, and lingual elastics tip the posterior teeth buccally with distobuccal rotation. Finally, mesiolingual elastics are recommended to minimize the excessive mesial tipping of posterior teeth.

## Data Availability

The datasets used and/or analyzed during the current study are available from the corresponding author on reasonable request.

## Abbreviations

3D: Three-dimensional
FEM: Finite element method
CA: Clear aligner
FA: Fixed appliances
FEA: Finite element analysis
PDL: Periodontal ligament
